# Long coverage with drug-eluting stents is superior to spot coverage for long femoropopliteal artery disease: PARADE II study

**DOI:** 10.3389/fcvm.2022.1022071

**Published:** 2022-10-19

**Authors:** Jong-Il Park, Young-Guk Ko, Yong-Joon Lee, Seung-Jun Lee, Sung-Jin Hong, Chul-Min Ahn, Jung-Sun Kim, Byeong-Keuk Kim, Myeong-Ki Hong, Cheol-Woong Yu, Seung-Woon Rha, Jong-Kwan Park, Pil-Ki Min, Chang-Hwan Yoon, Sang-Rok Lee, Sang-Ho Park, Dong-Hoon Choi

**Affiliations:** ^1^Division of Cardiology, Severance Cardiovascular Hospital, Yonsei University College of Medicine, Seoul, South Korea; ^2^Division of Cardiology, Korea University Anam Hospital, Seoul, South Korea; ^3^Division of Cardiology, Korea University Guro Hospital, Seoul, South Korea; ^4^Division of Cardiology, National Health Insurance Service Ilsan Hospital, Gyeonggi-do, South Korea; ^5^Division of Cardiology, Yonsei University Gangnam Severance Hospital, Seoul, South Korea; ^6^Division of Cardiology, Seoul National University Bundang Hospital, Seongnam-si, South Korea; ^7^Division of Cardiology, Jeonbuk National University Hospital, Jeonju-si, South Korea; ^8^Division of Cardiology, Soonchunhyang University Cheonan Hospital, Cheonan-si, South Korea

**Keywords:** peripheral artery disease, femoropopliteal artery, drug-eluting stent, patency, restenosis

## Abstract

**Background:**

The efficacy of spot stenting using drug-eluting stents (DES) for the treatment of long femoropopliteal (FP) lesion is unknown. This study aimed to compare clinical outcomes of long full coverage vs. spot coverage with DES for long FP artery disease.

**Methods:**

This multicenter randomized trial compared long DES vs. spot DES for FP lesions longer than 150 mm. All lesions were treated with paclitaxel-eluting stents (Zilver PTX). The primary endpoint was primary patency at 12 months.

**Results:**

The study was terminated early after an interim analysis. A total of 103 patients (55 in the long DES group; 48 in the spot DES group) were eligible for analysis. There were no significant differences in baseline and lesion characteristics between groups. Total stent length was longer in the long DES group than in the spot DES group (225.6 ± 67.2 vs. 131.3 ± 48.7 mm, *p* < 0.001). Technical success was achieved in all patients. There was a trend toward a higher primary patency rate at 12 months in the long DES group than in the spot DES group (87.5% vs. 67.5%, *p* = 0.120). The rate of survival free from target lesion revascularization was significantly higher in the long DES group than in the spot DES group (91.7% vs. 72.0%, *p* = 0.044). In multivariate Cox regression analysis, spot DES [hazard ratio (HR) 2.42, 95% confidence interval (CI) 1.14–5.12, *p* = 0.021] and postdilation (HR 0.27, 95% CI 0.09–0.79, *p* = 0.018) were identified as independent predictors for loss of patency at 12 months post-procedure.

**Conclusions:**

Long DES were more effective than spot DES for treating long FP lesions.

**Clinical trial registration:**

Clinicaltrials.gov, identifier: NCT02701881.

## Introduction

Self-expandable nitinol stents have higher patency rates than balloon angioplasty in femoropopliteal (FP) artery lesions (1, 2). However, restenosis after stenting occurs in 16 to 37% of patients by 1 year as the stent length increases, and this remains a major limitation of bare nitinol stents (3, 4). To reduce restenosis after stenting, drug-eluting stents (DES) were developed and adopted for the treatment of FP lesions. The Zilver PTX Drug-Eluting Stent (Cook Medical, Bloomington, IN, USA) is a self-expanding nitinol stent with a polymer-free paclitaxel coating designed to deliver paclitaxel locally to the vessel wall (5). Almost all of the paclitaxel is released within 72 h and remains in the vascular tissue for up to 56 days (5).

A randomized controlled clinical trial and various registry studies have reported superior efficacy of Zilver PTX, compared with balloon angioplasty or bare nitinol stents (6, 7). However, in FP lesions longer than 150 mm, the rate of restenosis after Zilver PTX implantation is as high as 37% by 1 year (8). The 1-year restenosis rate after full metal jacket stenting using Zilver PTX in lesions longer than 200 mm has been reported as 40%.

In previous studies using bare nitinol stents, spot stenting resulted in more favorable outcomes than long stenting (9, 10). However, the efficacy of spot stenting using DES in long FP lesions is unknown. Thus, the present study was designed to investigate clinical outcomes of long stenting vs. spot stenting using Zilver PTX for the treatment of long FP artery disease.

## Materials and methods

### Study design

The PARADE II (Comparison of the Primary Long vs. Short Coverage with Drug-Eluting Stents for Long Femoropopliteal Artery Disease II) study was a multicenter randomized controlled clinical trial comparing long DES implantation with full lesion coverage vs. spot DES implantation in patients with symptomatic FP artery disease. The major inclusion criteria were intermittent claudication or symptoms of critical limb-threatening ischemia (CLTI, Rutherford categories 2–5), FP artery lesions with stenosis > 50% and/or lesion length > 150 mm, and at least 1 patent runoff vessel. The major exclusion criteria were age > 85 years, severe CLTI (Rutherford category 6), acute limb ischemia, previous bypass surgery or stenting of the target FP artery, untreated inflow disease of the ipsilateral pelvic arteries (> 50% stenosis or occlusion), diseased distal popliteal artery (P2 or P3 segment) with stenosis > 50%, or a major bleeding event within the previous 2 months.

Based on the sample size calculation, the study was designed to enroll a total of 220 participants, randomized in a 1:1 manner. However, we decided to discontinue study enrollment in August 2019 because interim analysis showed a clinically relevant difference in outcomes between the study groups; the data and safety monitoring board for this study suggested early termination at that time. The trial protocol was approved by the local institutional review board of each participating center and was registered at www.clinicaltrials.gov (NCT02701881).

### Interventions

For all procedures, the patients received local anesthesia, which was supplemented with intravenous sedation and analgesia when required. Either ipsilateral or contralateral femoral puncture was performed, depending on the location of the target lesion. A 6F or 7F short introducer sheath (Terumo, Tokyo, Japan) was used for the ipsilateral approach, and a 6F to 7F long sheath (Balkin or Ansel; Cook Inc., Bloomington, IN, USA) was employed for the crossover approach. After the guidewire was passed through the target lesion, web-based randomization was performed. Patients were stratified according to the enrolling site and severity of ischemic symptoms (claudication vs. CTLI) and randomized to either the long stenting or spot stenting group. In cases of total occlusion, both intraluminal and subintimal approaches for recanalization were permitted. In both groups, predilation of the target lesion was performed prior to stent implantation. In the long DES group, stents were implanted to extend 10 mm proximally and distally from the margins of the target lesion with a luminal narrowing of > 50%. When multiple stents were required, margins of the stents overlapped by at least 10 mm. In the spot DES group, stents were implanted only in those segments with residual stenosis > 30% or a flow-limiting dissection after repeated predilation. In cases of an optimal result after predilation (without significant residual stenosis or flow-limiting dissection), a single stent was implanted to cover the most stenotic segment or the proximal stump of the occlusion before predilation. In both groups, Zilver PTX with a diameter of 5–7 mm was used for stenting at the FP target lesions. Postdilation with an up to 10% oversized balloon was performed when the residual stenosis was > 30%. After the procedure, aspirin (100 mg/day) was maintained indefinitely, and clopidogrel (75 mg/day) was prescribed for at least 6 months.

### Follow-up

We followed the patients clinically at 1, 3, 6, 9, 12, and 24 months after the procedure, according to the study schedule. Ankle-brachial index (ABI) was obtained at hospital discharge and at 6, 12, and 24 months post-procedure. An imaging study, such as intra-arterial angiography, computed tomography angiography (CTA), or duplex ultrasound, was performed at 12 months or if there was a > 0.15 decrement in ABI or worsening symptoms, as reflected by a change in Rutherford category.

### Study endpoints and definitions

Technical success was defined as recanalization of the target lesion, with residual stenosis of 30% or less and no flow-limiting dissection. The primary endpoint was the primary patency at 12 months after the procedure, according to the stenting strategy. Primary patency was defined as treated FP lesions without > 50% restenosis, as assessed by an imaging study (intra-arterial angiography, CTA, or duplex ultrasound). A lesion/adjacent segment velocity ratio > 2.4 by duplex was considered indicative of > 50% restenosis (11). The secondary endpoint was freedom from clinically driven target lesion revascularization (TLR). Clinically driven TLR was performed for restenotic lesions with both worsening symptoms and a > 0.15 decrement in ABI. Major complications were defined as any event that was either fatal or required surgical treatment or re-hospitalization within 30 days after the procedure.

### Statistical analysis

Continuous data are presented as mean ± standard deviation, and categorical data are presented as count (percentage). Patient, lesion, and procedural data were compared between the 2 groups using the Fisher exact test or χ2 test for categorical data or the Student's *t*-test for continuous data. We estimated the primary and secondary endpoints using Kaplan-Meier survival analysis and compared the results of the two groups using the log-rank test. We performed univariate analysis using Cox proportional hazards regression to identify potential risk factors (clinical and procedural variables) for restenosis at 12 months post-procedure. Variables achieving a *p*-value < 0.15 in univariate analysis were entered into the multivariate analysis. *P*-values < 0.05 were considered statistically significant. All statistical analyses were performed using SPSS (version 25.0; IBM, Chicago, IL, USA).

## Results

### Baseline clinical data

**Figure 1:**
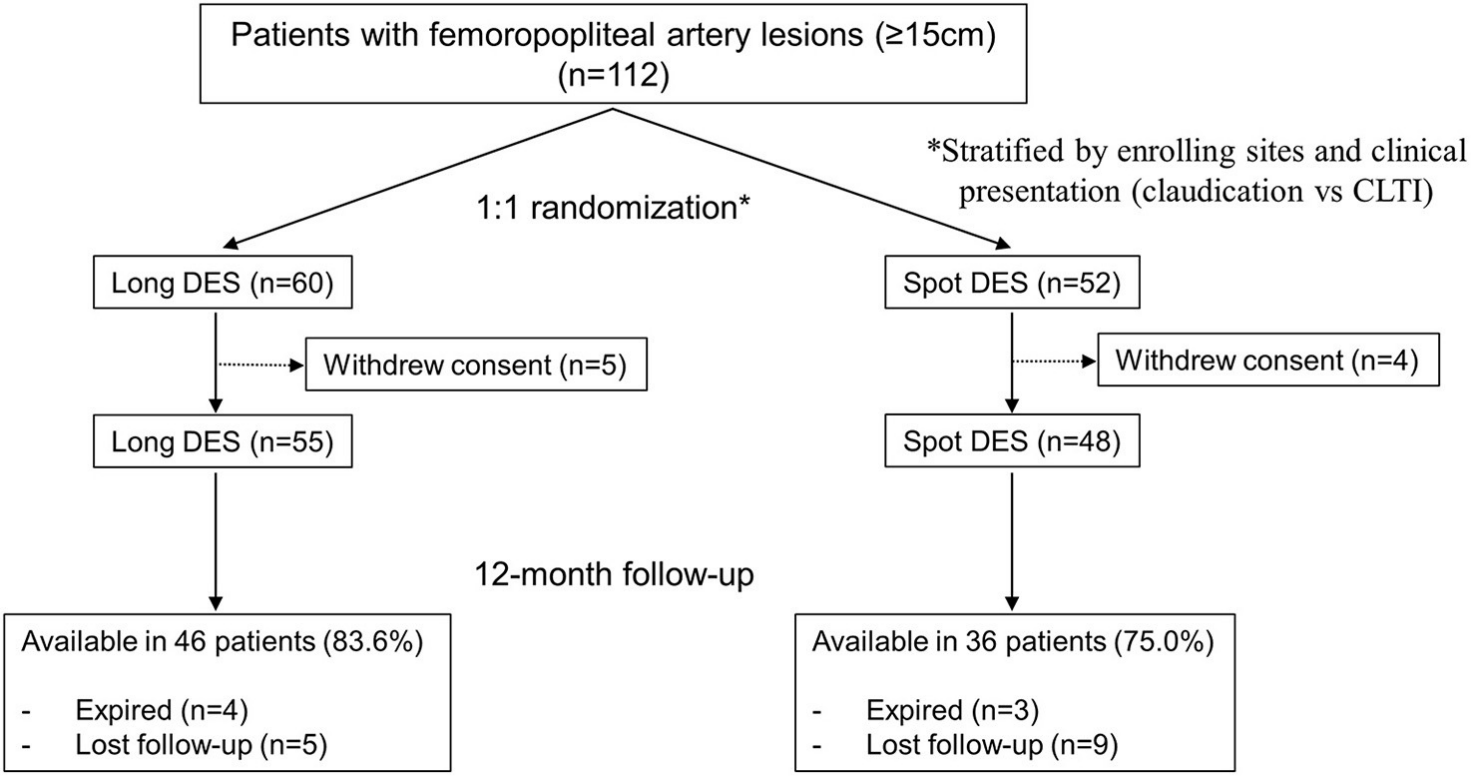
Patient flow diagram. CLTI, chronic limb-threatening ischemia; DES, drug-eluting stents.

From January 2016 through May 2019, a total of 112 patients were enrolled in this study and randomized to either the long DES group or spot DES group. After excluding 9 patients because of inclusion and exclusion criteria violations, 103 patients (55 in the long DES group and 48 in the spot DES group) were included in the final analysis, as shown in Figure 1.

Baseline clinical characteristics are summarized in Table 1. When considering all 103 patients, the mean patient age was 70.9 ± 8.6 years, and the majority (82.5%) of study participants were male. Diabetes mellitus and chronic kidney disease were present in 61.2 and 34.0% of patients, respectively. CLTI was present in 27.2% of participants. Baseline clinical characteristics did not differ significantly between groups. Medications at discharge also did not differ between groups, except for anticoagulants (warfarin or direct oral anticoagulants), which were prescribed more frequently in the spot DES group (0% vs. 8.3%, *p* = 0.044).

**Table 1:** Baseline clinical characteristics.

**Characteristic**	**Long DES**	**Spot DES**	***P*-value**
	**(*n* = 55)**	**(*n* = 48)**	
Age (y)	70.8 ± 8.3	71.1 ± 9.2	0.861
Male	47 (85.5)	38 (79.2)	0.402
BMI (kg/m^2^)	23.3 ± 3.2	23.0 ± 3.4	0.599
Hypertension	41 (74.5)	37 (77.1)	0.764
Diabetes mellitus	32 (58.2)	31 (64.6)	0.506
Hypercholesterolemia (TC > 200 mg/dL)	18 (32.7)	17 (35.4)	0.458
Chronic kidney disease	13 (23.6)	10 (20.8)	0.917
Hemodialysis	7 (12.7)	4 (8.3)	0.761
Coronary artery disease	29 (52.7)	28 (58.3)	0.568
Current smoker	16 (29.1)	17 (35.4)	0.635
Previous stroke	6 (10.9)	9 (18.8)	0.260
CLTI	17 (31.5)	11 (22.9)	0.333
**Medication at discharge**			
Aspirin	50 (90.9)	44 (91.7)	0.892
Clopidogrel	50 (90.9)	43 (89.6)	0.821
Cilostazol	21 (38.2)	15 (31.3)	0.462
Warfarin/DOAC	0 (0)	4 (8.3)	0.044
Statin	44 (80.0)	38 (79.2)	0.917

Data are mean ± standard deviation or count (percentage).

BMI, body mass index; CLTI, chronic limb-threatening ischemia; DES, drug-eluting stents; DOAC, direct oral anticoagulant; TC, total cholesterol.

### Lesion and procedural data

Lesion and procedural characteristics are presented in Table 2. When considering all 103 patients, the mean lesion length was 242.7 ± 64.5 mm. Total occlusion and severely calcified lesions were present in 78.6 and 17.5% of participants, respectively. Lesion length and rates of total occlusion, severely calcified lesions, and Trans-Atlantic Inter-Society Consensus (TASC) II lesion types did not differ significantly between groups.

**Table 2:** Lesion and procedural data.

**Variable**	**Long DES**	**Spot DES**	***P*-value**
	**(*n* = 55)**	**(*n* = 48)**	
TASC II lesion type			0.338
B	2 (3.6)	5 (10.4)	
C	42 (76.4)	36 (75.0)	
D	11 (20.0)	7 (14.6)	
Lesion length (mm)	245.1 ± 58.7	238.6 ± 63.7	0.597
Total occlusion	46 (85.2)	35 (72.9)	0.126
Severe calcification	10 (18.2)	8 (16.7)	0.840
**Combined treatment**			
Iliac lesion	7 (12.7)	9 (18.8)	0.400
BTK lesion	7 (12.7)	8 (16.7)	0.572
Subintimal approach	28 (50.9)	26 (54.2)	0.741
Atherectomy	2 (3.6)	0 (0)	0.497
Number of implanted stents	2.5 ± 0.8	1.5 ± 0.6	0.024
Total stented length (mm)	225.6 ± 67.2	131.3 ± 48.7	< 0.001
Full lesion coverage with stents	55 (100.0)	7 (14.6)	< 0.001
Postdilation	10 (18.2)	16 (34.0)	0.067
Technical success	55 (100.0)	48 (100.0)	1.000
Pre-procedure ABI	0.62 ± 0.23	0.55 ± 0.21	0.178
Post-procedure ABI	0.87 ± 0.17	0.84 ± 0.19	0.507
**Complications**			
Major	0 (0)	1 (2.1)	0.466
Access site bleeding	0 (0)	2 (4.2)	0.215
Vascular perforation	0 (0)	0 (0)	-
Distal embolization	1 (1.8)	0 (0)	1.000

Data are mean ± standard deviation or count (percentage).

ABI, ankle-brachial index; BTK, below-the-knee; DES, drug-eluting stents; TASC, Trans-Atlantic Inter-Society Consensus.

Procedural success was achieved in all patients. However, 7 patients (14.6%) in the spot DES group underwent unplanned full-lesion stent coverage because of severe dissection. The number of implanted stents was significantly higher (2.5 ± 0.8 vs. 1.5 ± 0.6, *p* = 0.024) and the total stented length was significantly longer (225.6 ± 67.2 mm vs. 131.3 ± 48.7 mm, *p* < 0.001) in the long DES group than in the spot DES group. In addition, there was a trend toward more frequent postdilation performed in the short DES group than in the long DES group (34% vs. 18.2%, *p* = 0.067). Atherectomy using Turbohawk (Medtronic, Santa Rosa, CA, USA) was performed in two patients of the long DES group. Embolic protection device was not utilized in this study.

ABI was not significantly different between the two groups before or after the procedure. There were 2 cases of access site bleeding in the spot DES group, 1 of which required surgical repair and was thus considered a major complication. There was 1 case of distal embolization in the long DES group, which was managed with endovascular embolectomy.

### Clinical outcomes

The mean duration of clinical follow-up was 535.8 ± 251.2 days. A total of 84 patients completed 1 year of follow-up: 46 in the long DES group and 38 in the spot DES group (Figure 1). In the long DES group, there were 4 deaths (2 from a cardiovascular cause; 2 from an unknown cause) and 5 cases of loss to follow-up. In the spot DES group, there were 3 deaths (1 from a non-cardiovascular cause; 2 from an unknown cause) and 9 cases of loss to follow-up.

**Figure 2:**
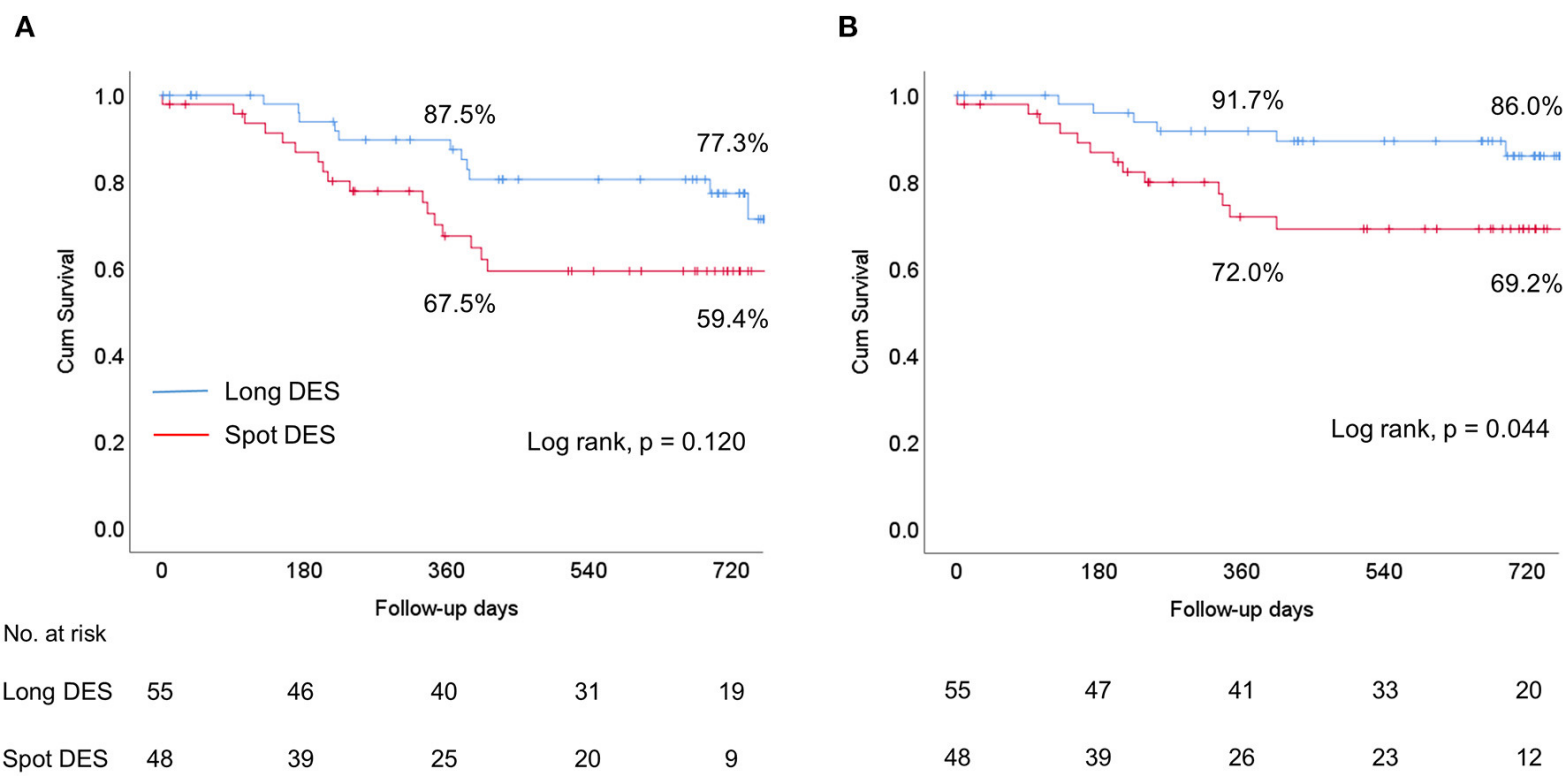
Kaplan-Meier survival curves comparing the long drug-eluting stent (DES) group vs. the spot DES group. **(A)** Primary patency. **(B)** Target lesion revascularization (TLR)-free survival. Cum, cumulative.

There was a trend toward higher primary patency in the long DES group than in the spot DES group (Figure 2A). Specifically, the primary patency rates were 87.5% at 12 months and 77.3% at 24 months in the long DES group and 67.5% at 12 months and 59.4% at 24 months in the spot DES group (*p* = 0.120). Survival free from TLR was significantly higher in the long DES group than in the spot DES group (Figure 2B). Specifically, the TLR-free survival rates were 91.7% at 12 months and 86.0% at 24 months in the long DES group and 72.0% at 12 months and 69.2% at 24 months in the spot DES group (*p* = 0.044). In multivariate Cox regression analysis, spot DES [hazard ratio (HR) 2.42, 95% confidence interval (CI) 1.14–5.12, *p* = 0.021] and postdilation (HR 0.27, 95% CI 0.09–0.79, *p* = 0.018) were identified as independent predictors for loss of patency at 12 months post-procedure (Table 3).

**Table 3:** Predictors of loss of patency at 12 months in Cox proportional hazards regression analysis.

**Factor**	**Univariate analysis**		**Multivariate analysis**	
	**HR (95% CI)**	***P*-value**	**HR (95% CI)**	***P*-value**
Age	0.98 (0.94–1.02)	0.279		
Male	1.11 (0.42–2.59)	0.829		
Body mass index	1.04 (0.93–1.17)	0.448		
Hypertension	1.21 (0.52–2.84)	0.658		
Diabetes mellitus	1.82 (0.82–4.01)	0.140	1.76 (0.77–4.03)	0.180
Hypercholesterolemia	1.20 (0.55–2.62)	0.652		
Current smokers	1.43 (0.67–3.04)	0.361		
Chronic kidney disease	1.27 (0.59–2.74)	0.538		
Hemodialysis	2.70 (1.02–7.18)	0.046	2.22 (0.80–6.12)	0.124
Coronary artery disease	1.06 (0.51–2.19)	0.872		
Previous stroke	1.24 (0.43–3.58)	0.694		
CLTI	0.87 (0.35–2.14)	0.753		
Pre-procedure ABI	0.66 (0.10–4.50)	0.669		
Lesion length (per 10 mm)	0.97 (0.91–1.04)	0.417		
Total occlusion	1.10 (0.42–2.90)	0.850		
TASC II D lesion	1.50 (0.64–3.53)	0.354		
Subintimal approach	1.08 (0.51–2.29)	0.842		
Combined iliac intervention	0.59 (0.18–1.97)	0.393		
Combined BTK intervention	1.00 (0.35–2.89)	0.997		
Spot DES	1.76 (0.85–3.64)	0.125	2.42 (1.14–5.12)	0.021
Postdilation	0.37 (0.13-1.06)	0.065	0.27 (0.09-0.79)	0.018
Oral anticoagulants	1.57 (0.55–4.47)	0.397		

ABI, ankle-brachial index; BTK, below-the-knee; CI, confidence interval; CLTI, chronic limb-threatening ischemia; DES, drug-eluting stents; HR, hazard ratio; TASC, Trans-Atlantic Inter-Society Consensus.

## Discussion

The main findings of this study were that the long DES strategy was more effective than the spot DES approach for endovascular treatment of long FP lesions in terms of primary patency and freedom from TLR.

Zilver PTX, a paclitaxel-eluting stent, has shown excellent 12-month primary patency rates ranging from 86.4 to 90.4% in clinical trials with mean FP lesion lengths < 150 mm. However, there have been only a few studies investigating the efficacy of Zliver PTX in very long FP lesions (> 200 mm). A substudy of the Zilver PTX single-arm study involving lesions with a mean length of 226 mm demonstrated a primary patency rate of 77.6% and TLR-free survival rate of 88% at 12 months (12). Other studies reported 12-month primary patency rates ranging from 60 to 74.5% and TLR-free survival rates of 79% and 85.4% for very long lesions (13, 14). Thus, primary patency rates of DES appear to decrease with increasing lesion length.

In the current study, the mean lesion length of the study population was 242 mm, and the primary patency rates for the entire population were 78.0% at 12 months and 68.8% at 24 months. These rates are generally comparable to those of previous studies. However, we found that the primary patency rate at 12 months was well maintained at 87.5% in the long stent group but fell to 67.5% in the spot stent group. There have been only a few studies that reported on the outcomes of spot stenting. In a retrospective study, Tomoi et al. showed a lower 3-year patency rate with spot stenting compared with full coverage stenting for FP lesions (15). However, this study used not only DES but also bare-metal nitinol stent. The study concluded that spot stenting was non-inferior to full coverage stenting for primary patency at lesion length ≥138 mm. By contrast, Iida et al. found in a prospective single-arm study using fluoropolymer-based DES study (Eluvia, Boston Scientific) that spot stenting was associated with an increased risk of 12-month restenosis with odds ratio of 2.44 (16). Whether full jacket stenting was performed in the previous studies is unknown, except for the small retrospective study by Phillips et al. (14). In their study, the 12-month primary patency of full jacket Zilver PTX stenting for lesions > 200 mm was only 60%. Differences in various factors (e.g., baseline clinical and lesion characteristics, medications) may have contributed to discrepancies in results between the present study and previous studies. In particular, differences in the inclusion of distal popliteal artery lesions and in-stent restenosis might have led to varying reported outcomes for Zilver PTX. Stenting in the distal popliteal artery is known to be associated with an increased risk of restenosis (13, 17), and in the present study, we excluded patients with FP lesions involving P2 or P3 popliteal artery segments. By contrast, in the study of Phillips et al., all lesions > 200 mm were TASC II D lesions; thus, it is likely that a large proportion of their study subjects had distal popliteal artery involvement. Additionally, in-stent restenosis accounted for 14.4%−29% of lesions in previous studies, whereas we excluded all patients with in-stent restenosis. Repeated endovascular treatment of in-stent restenosis lesions is associated with generally poorer outcomes than treatment of *de-novo* lesions (18). Our group previously reported that using bare nitinol stents, spot stenting achieved better outcomes than long stenting in the treatment of long FP lesions (9, 10). Therefore, our current results appear to contradict our prior findings. However, there are two principal differences between the present and previous studies. First, DES were used in all patients in the current study. Second, lesions with distal popliteal artery involvement were excluded in the present study but were included in our previous studies. Compared to bare metal stents, DES have been shown to more effectively inhibit neointimal proliferation in coronary and peripheral arteries and were able to reduce the restenosis rate, even when long “full jacket” stents were implanted (7, 19, 20). However, when DES were implanted in a segment shorter than the total lesion length, there was a higher possibility of restenosis in the non-stented segment treated with balloon angioplasty alone. Furthermore, the stent border zones that were injured by balloon angioplasty but not covered by DES exhibited more active neointimal proliferation and an increased risk of restenosis (21, 22). This phenomenon, known as a geographic miss, has been described as an important factor leading to stent failure in coronary artery interventions and likely accounts for the poorer results with spot stenting in this study. Based on the present study results, spot stenting using DES after plain balloon angioplasty appears to be inappropriate. However, provisional spot stenting after drug-coated balloon (DCB) has shown to be effective according to clinical trials on DCBs for FP artery lesions (23, 24). Thus, it would be interesting to compare DCB with spot stenting vs. full metal jacket stenting using DES in the future studies.

This study has several limitations. First, it was underpowered because of insufficient subject enrollment. This was the consequence of the early termination of the study, which was recommended by the data and safety monitoring board because interim analysis revealed a clinically relevant difference in outcomes between study groups. Second, spot stenting was defined arbitrarily. We did not set a limit on the stented length or stent-to-lesion length ratio. Third, we did not routinely perform intravascular ultrasound during the procedure to verify subintimal passage of the wires. Fourth, the quality of life before and after the intervention was not investigated because there were many elderly patients who could not give an appropriate answer to the questionnaire due to their comorbidities.

## Conclusions

Long DES was more effective for treating diffuse long FP lesions than spot DES in terms of primary patency and freedom from TLR. These results suggest that when treating FP disease with DES, lesions should be fully covered by these stents to achieve better outcomes.

## Data availability statement

The original contributions presented in the study are included in the article/supplementary material, further inquiries can be directed to the corresponding authors.

## Author contributions

Y-GK and D-HC: conception, design, and overall responsibility. J-IP and Y-GK: analysis, interpretation, and writing the article. Y-GK, D-HC, Y-JL, S-JL, S-JH, C-MA, J-SK, B-KK, M-KH, C-WY, S-WR, J-KP, P-KM, C-HY, S-RL, and S-HP: data collection. All authors contributed to the article and approved the submitted version.

## Funding

This study was supported by grants from Cook Medical Korea; the Korea Health Technology R&D Project through the Korea Health Industry Development Institute (KHIDI) (No. HI20C1566) and the Bio & Medical Technology Development Program of the National Research Foundation & MSIT (No.2020M3A9I4038455); the Patient-Centered Clinical Research Coordinating Center (PACEN) funded by the Ministry of Health & Welfare, Republic of Korea (HC20C0081); and the Korea Medical Device Development Fund funded by the Korean government (202011B29-03, 202011D12-02) and the Cardiovascular Research Center (Seoul, Korea).

## Conflict of interest

Y-GK and D-HC received institutional research grants from Cook Medical, Medtronic, Boston Scientific, Samjin Pharm, Korea United Pharm, Dong-A Pharm, and Otsuka Korea. None of these companies, including Cook Medical, were involved in developing study protocols or study process of PARADE II. The remaining authors declare that the research was conducted in the absence of any commercial or financial relationships that could be construed as a potential conflict of interest.

## Publisher's note

All claims expressed in this article are solely those of the authors and do not necessarily represent those of their affiliated organizations, or those of the publisher, the editors and the reviewers. Any product that may be evaluated in this article, or claim that may be made by its manufacturer, is not guaranteed or endorsed by the publisher.

## Abbreviations

DES: drug-eluting stents
FP: femoropopliteal
TLR: target lesion revascularization
TASC: Trans-Atlantic Inter-Society Consensus
ABI: ankle-brachial index
CTA: computed tomography angiography
CLTI: chronic limb-threatening ischemia
PTA: percutaneous transluminal angioplasty.
